# KRAS: A Druggable Target in Colon Cancer Patients

**DOI:** 10.3390/ijms23084120

**Published:** 2022-04-08

**Authors:** Francesca Negri, Lorena Bottarelli, Gian Luigi de’Angelis, Letizia Gnetti

**Affiliations:** 1 Gastroenterology and Endoscopy Unit, Azienda Ospedaliero-Universitaria di Parma, 43126 Parma, Italy; gianluigi.deangelis@unipr.it; 2 Department of Medicine and Surgery, University of Parma, 43126 Parma, Italy; lorena.bottarelli@unipr.it; 3 Pathology Unit, Azienda Ospedaliero-Universitaria di Parma, 43126 Parma, Italy; lgnetti@ao.pr.it

**Keywords:** *KRAS* oncogene, RAS pathway, colon cancer, targeted therapy, sotorasib, adagrasib

## Abstract

Mutations in *KRAS* are among the most frequent aberrations in cancer, including colon cancer. KRAS direct targeting is daunting due to KRAS protein resistance to small molecule inhibition. Moreover, its elevated affinity to cellular guanosine triphosphate (GTP) has made the design of specific drugs challenging. Indeed, KRAS was considered ‘undruggable’. KRASG12C is the most commonly mutated variant of KRAS in non-small cell lung cancer. Currently, the achievements obtained with covalent inhibitors of this variant have given the possibility to assess the best therapeutic approach to KRAS-driven tumors. Mutation-related biochemical assets and the tissue of origin are expected to influence responses to treatment. Further attempts to obtain mutant-specific KRAS (KRASG12C) switch-II covalent inhibitors are ongoing and the results are promising. Drugs targeted to block KRAS effector pathways could be combined with direct KRAS inhibitors, immunotherapy or T cell-targeting approaches in KRAS-mutant tumors. The development of valuable combination regimens will be essential against potential mechanisms of resistance that may arise during treatment.

## 1. Introduction

The *KRAS* oncogene has been studied extensively in human malignancies since its discovery back in the 1960s [1,2]. Activating *KRAS* mutations are present in more than 20% of human cancers, even though *KRAS* mutation rates are dissimilar in different ethnicities and different colorectal cancer (CRC) locations [2].

Thorough attempts to identify the mechanisms causing intracellular protein trafficking, alterations and KRAS signaling pathways have suggested several potential therapeutic approaches [3]. Furthermore, *KRAS* mutation is one of the most important predictive markers in determining resistance to epidermal growth factor receptor (EGFR) inhibitors [4]. However, *KRAS* mutation is far from being simply an unfavorable predictive marker but is rather part of an evolving research process driven by an important clinical unmet need [3,4]. Nowadays, the chemotherapy options for the treatment of patients with advanced *KRAS* mutant microsatellite stable (MSS) CRC are cytotoxic doublets such as FOLFIRI, FOLFOX or FOLFOXIRI in combination with anti-vascular endothelial growth-factor (VEGF) drugs (i.e., bevacizumab and aflibercept) [5]. Thus, additional studies are required to clarify the processes responsible for KRAS signaling to evaluate distinct possible therapeutic strategies.

KRAS belongs to the family of the guanine nucleotide-binding proteins [1]. KRAS shifts between an inactive, guanosine diphosphate (GDP)-bound (“off”) state, and an active, guanosine triphosphate (GTP)-bound (“on”) state. While bound to GDP, KRAS is inactive. Receptor activation directs the activation of a family of guanine nucleotide exchange factors (GEFs), which trigger GDP’s exchange with GTP. GTP-bound KRAS transduces signals to downstream effectors and stimulates multiple signaling pathways [6,7], including the RAF family of kinases and phosphatidylinositol 3-kinase (PI3K), that in turn activate a cascade of events and influence a multitude of cellular processes, including cell proliferation, differentiation, survival, growth and apoptosis, all renowned hallmarks of cancer [8]. 

**Figure 1:**
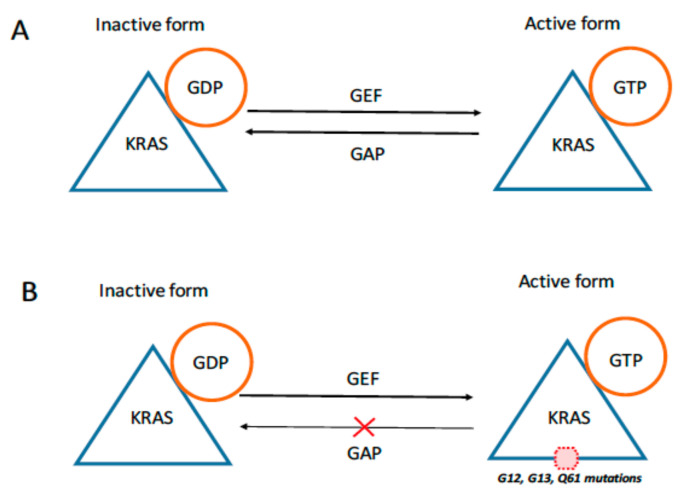
A schematic of KRAS signaling in normal (**A**) and tumoral (**B**) cells is shown. Mutant KRAS is permanently in a GTP-bound active state.

Normally, KRAS activation is transient since guanosine triphosphatase (GTPase), an endogenous nucleotidase, switches off the pathway by transforming RAS-GTP to RAS-GDP (Figure 1). The majority of *KRAS* somatic mutations impair GTPase activity and cause an increase in RAS-GTP levels that unrestrain RAS effectors and PI3K signaling and trigger the uncontrolled proliferation characteristic of malignant disorders [9,10]. Nearly all factors of the RAS pathway are altered in cancer, most frequently activating mutations of *KRAS*, *NRAS*, *HRAS*, *BRAF* and receptor tyrosine kinases (RTKs).

Various attempts to produce KRAS inhibitors have failed in the last decade. Ostrem et al. [11] described a pocket in the switch II effector region of a mutant allele, KRASG12C, and uncovered a compound, ARS-853, that prevents KRAS-GTP by a novel mechanism that depends on the lasting GTPase activity of KRASG12C. Recently, different KRASG12C inhibitors have been developed. Preclinical data indicate that they are KRASG12C-specific and should produce less side effects. The study of KRAS pathways will lead to the development of new molecules, but it might also unearth the potential of engineered CD8 in therapy.

This paper gives an overview of the latest advances and therapies targeting mutant KRAS and deals with combination approaches that could theoretically increase the clinical benefit of KRAS inhibition. We also provide a description of the RAS pathway, the main mechanisms involved in resistance and emerging therapeutic strategies to treat KRAS-mutant tumors.

## 2. The RAS Family

The RAS family consists of membrane-associated small GTPases that hydrolyze GTP to GDP and function as regulators of intracellular signaling transduction cascades involved in cell growth, differentiation and survival [12,13].

### 2.1. RAS Upstream Activators

RAS protein activity is modulated by receptor-tyrosine kinases (RTKs), G proteins, cytokines receptors and extracellular matrix receptors.

**Figure 2:**
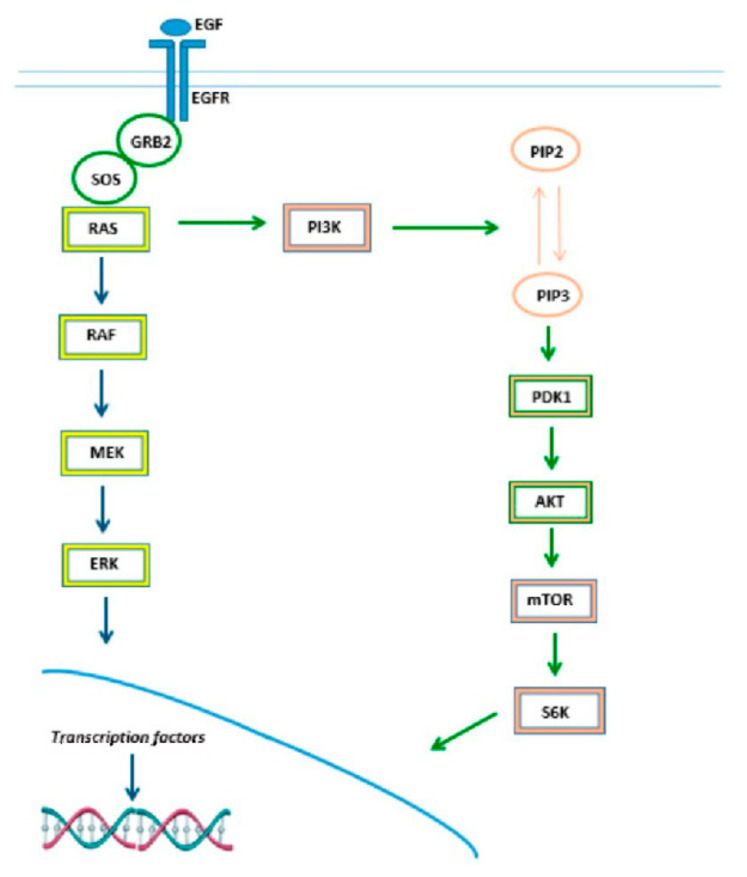
MAPK/ERK and PI3K/Akt/mTOR signaling pathway.

Many growth factors, such as epidermal growth factor (EGF), platelet-derived growth factor (PDGF) and fibroblast growth factors (FGFs), activate RTKs and then switch on RAS proteins with the help of different molecules. The most characterized pathway involves the interaction of EGF to its receptor EGFR. In particular, the ligand binding to EGFR induces dimerization of the receptor monomer and stimulates the activation of intrinsic tyrosine kinase and autophosphorylation. The phosphorylated receptor binds to an adaptor protein GRB2 (growth factor receptor-bound protein 2) and recruits the GEF SOS (son of sevenless) to the plasma membrane, which consequently transforms GDP-bound RAS to active GTP-bound RAS. Then, RAS proteins act as “molecular switches” and cycle between the GDP-bound (inactive) and GTP-bound (active) conformational states (Figure 1). The RAS GDP-GTP cycle is controlled by GEFs that promote nucleotide exchange and the formation of RAS-GTP. GTPase-activating proteins (GAPs) induce the hydrolysis of GTP, forming inactive RAS-GDP. Active RAS-GTP binds to various downstream effectors, which are mainly involved in the activation of the mitogen-activated protein kinase (MAPK) and PI3K pathways (Figure 2) [14].

### 2.2. RAS Downstream Effectors: MAPK/ERK and PI3K/Akt/mTOR Signaling Pathway

The first described RAS-effector was the MAPK/ERK (extracellular signal-regulated kinases) signaling cascade; it regulates a variety of normal cellular functions, such as cell growth, differentiation, inflammation and apoptosis. Following RAS activation, a complex series of processes including phosphorylation and dimerization activate the serine/threonine kinase RAF (A-RAF, B-RAF and C-RAF). Activated RAF phosphorylates MEK (Mitogen-Activated Protein Kinase), thus leading to downstream ERK phosphorylation. Once phosphorylated, ERK1/2 translocates into the nucleus, where it activates c-JUN and c-FOS, two key transcription factors of the AP-1 family. These factors induce the transcription of genes involved in cellular processes and cell cycle progression [15,16].

The PI3K pathway is the second best-characterized RAS-effector and participates in the regulation of cell growth, cell cycle entry, cell survival, cytoskeleton reorganization, and metabolism. When active, PI3K transforms phosphatidylinositol (4,5)-bisphosphate (PIP2) into phosphatidylinositol (3,4,5)-trisphosphate (PIP3). PIP3 propagates intracellular signaling, which brings phosphoinositide-dependent kinase 1 (PDK1) and AKT to the membrane. Once activated, AKT modulates cell cycle progression and survival by phosphorylating mTOR (serine/threonine kinase mammalian target of rapamycin), which functions as two distinct multiprotein complexes, RAPTOR-associated mTOR complex 1 (mTORC1) and RICTOR-associated mTOR complex 2 (mTORC2). The Rheb GTPase-mediated activation of mTORC1 results in the phosphorylation of ribosomal protein S6 kinase and the translational repressor protein 4E-BP1 finally regulating different cellular processes such as protein synthesis, autophagy and cell aging [17].

## 3. Type and Frequency of *KRAS* Mutation

The three *RAS* oncogenes (*HRAS*, *KRAS* and *NRAS*) are part of the most frequently mutated gene family in human cancer, since over 20% of human cancers harbor mutations in one of the three *RAS* genes, making them the most prevalent oncogenic drivers [13]. They are mutated at different prevalence rates in human cancers [13,18,19]. *KRAS* is the most frequently mutated (85% of all RAS-driven cancers), followed by *NRAS* (12%) and *HRAS* (3%) (COSMIC v80) [20]. In particular, the prevalence of *KRAS* mutations is about 30% in non-small cell lung cancer (NSCLC), 30–50% in CRC, 80% in pancreatic adenocarcinoma and 45–54% in extrahepatic cholangiocarcinoma [21].

*KRAS* codon 12, 13, 61 and 146 specific point mutations occur frequently in *KRAS*-mutated cancers. They are clustered in exon 2 (codons 12 and 13), exon 3 (codon 61) or exon 4 (codon 146) and maintain the KRAS-GTP active form and consequently result in tumor initiation and progression. In particular, more than 90% of *KRAS* mutations occur at glycine 12. This amino acid lies in the P-loop region of the protein and plays a key role in stabilizing nucleotide binding [22,23].

The relationship between *KRAS* mutation and CRC was postulated for the first time by Fearon and Vogelstein in 1990 [24]. They described the model of carcinogenesis as a multistep process involving cumulative genetic mutations. KRAS oncogenic activation is crucial for the malignant transformation of the colonic epithelium. Approximately 30–50% of CRCs develop *KRAS* mutations as an early event [25,26].

Roughly 3000 *KRAS* point mutations have been reported in CRC (www.sanger.ac.uk/genetics/CGP/cosmic/, accessed on 10 February 2022). The activating point mutation of the *KRAS* oncogene at codon 12 (exon 2) is the initiating event in the majority of CRC cases (83%) [27,28]. Mutations at codons 13, 61 and 146 contribute to a lesser degree, accounting for ~17%, ~2% and ~4%, respectively. The most clinically frequent substitutions are aspartate (G12D, 36%) followed by valine (G12V, 23%), both of which are found to occur at codon 12. These allelic mutations are located in close proximity to the GTP-binding site, thus interfering with GTPase activity by impairing the GAP-mediated hydrolysis of GTP to GDP, thereby locking the KRAS protein in a hyperexcitable state [20]. With regard to rectal tumors, *KRAS* mutations are detected in ∼35–45% of patients with local advanced rectal cancer and KRAS status shows no difference between colon cancer and rectal cancer [29,30].

The *KRAS* mutational status emerged as clinically relevant, since wild-type profile predicts early response to anti-EGFR monoclonal antibodies therapy and *KRAS* somatic mutations at codons 12 and 13 were identified as being associated with a lack of response [31,32]. Eventually, other hotspot mutations, *KRAS* exons 3 and 4 (comprising codons 59–61 and 117–146, respectively) and *NRAS* exons 2, 3 and 4 (comprising codons 12–13, 59–61 and 117–146, respectively), were found to be associated with clinical resistance to anti-EGFR treatment [23]. Thus, validation of *RAS* wild-type status is mandatory to establish treatment in advanced CRC [30] and extended *RAS* testing investigating exons 2, 3 and 4 (codons 12, 13, 59, 61, 117 and 146) should be included, as only patients showing pan-*RAS* wild-type status should be treated with EGFR inhibitors according to current guidelines [5].

Polymerase chain reaction (PCR)-based assays are usually employed to investigate the *KRAS* and *NRAS* mutational status at the main hotspot mutation. On the contrary, next-generation sequencing (NGS) assays are able to detect uncommon mutational profiles, allowing the analysis of full exons. The impact of non-hotspot mutations of *KRAS* and *NRAS* on anti-EGFR monoclonal antibody resistance is still unclear and it may be useful to study their effects in patients with metastatic CRC [33].

## 4. KRAS-Targeted Therapies

**Figure 3:**
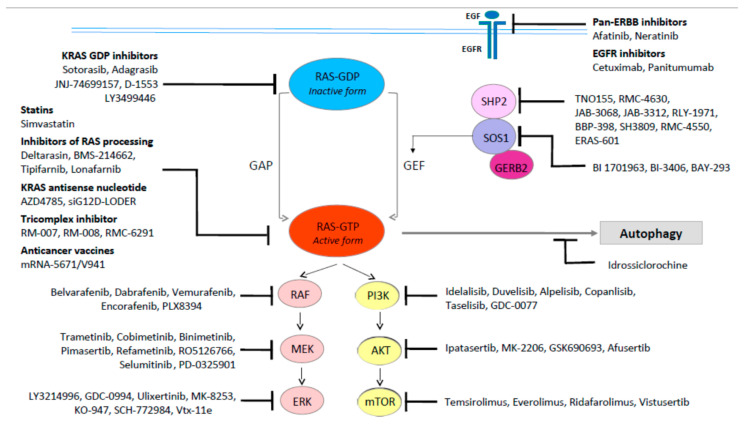
KRAS-targeting strategies.

The development of valuable drugs to prevent RAS-driven oncogenesis was challenging for more than three decades and RAS was deemed ‘undruggable’. Yet, sotorasib (AMG 510), an allele-specific covalent KRAS inhibitor, was recently approved by the FDA [34]. Sotorasib binds to KRASG12C, the third most common mutation at this locus. In addition, adagrasib (MRTX 849), a potent, covalent KRAS G12C inhibitor, is also being assessed by the FDA for accelerated approval as a treatment for patients with previously treated KRASG12C mutant NSCLC [35]. KRASG12C mutations predominate in NSCLC including 11–16% of lung adenocarcinomas (45–50% of mutant KRAS is G12C) and about 3% of CRC [36]. The KRASG12C mutation leads to glycine replacement with cysteine at position 12 [37]. The effective inhibition of KRASG12C has paved the way for a wide range of mutant RAS allele-specific targeted therapies. Further approaches to inhibit this pathway are being explored, such as the inhibition of SOS1 and SHP2, which foster RAS activation, and the inhibition of RAS downstream pathways [38] (Figure 3). Specifically, different therapeutic approaches targeting MAPK/ERK and PI3K/Akt/mTOR signaling, metabolic pathways, or hampering KRAS through immunotherapy combinations have already been tested or are being tested in RAS mutant CRC. Mutation-specific biochemical assets, the presence of co-mutations, and the tissue of origin are likely to influence treatment effectiveness.

### 4.1. Strategies to Target KRAS Directly

RAS direct inhibition is an attractive approach for treating RAS-mutant cancers (Figure 3). Shokat et al. [38] originally characterized an allosteric binding pocket behind switch-II, designated the switch-II pocket, in the mutant KRASG12C protein. The G12 codon is a mutational hotspot in KRAS; the intrinsically reactive nature of cysteine, located at codon 12, can be used to produce covalent small-molecule inhibitors [38]. Notably, wild-type KRAS does not have cysteines in the active site; therefore, KRASG12C can be specifically inhibited. Huge efforts to target RAS directly have been made, involving the development of switch-II mutant-selective covalent inhibitors and pan-RAS inhibitors.

Potent KRASG12C inhibitors are currently in clinical trials, even though few results have been issued (Table 1). In early-phase clinical trials, two potent, selective and irreversible small-molecule KRASG12C inhibitors, sotorasib and adagrasib, have shown encouraging results in NSCLC, but limited activity in CRC [39,40,41]. Sotorasib has shown an objective response rate (RR) of 37.1% among NSCLC patients and a RR of less than 10% among CRC patients [39]. Preliminary results of phase 1/2 trials of adagrasib involving patients with KRASG12C-mutant cancer have also shown a higher objective RR amongst NSCLC patients than CRC patients (45% vs. 17%, respectively) [40,41,42]. More data are required to ascertain if there is a difference related to tumor biology [41,42].

KRASG12C inhibitors retain synergistic growth-inhibitory activity when combined with inhibitors of proteins that stimulate or are triggered by RAS, i.e., AKT, MEK, PI3K, proteins of the EGFR pathway or immunotherapy [43,44]. As part of the KRYSTAL-1 phase 1/2 trial, adagrasib was investigated as a monotherapy and in combination with cetuximab in a series of heavily pre-treated patients with KRASG12C-mutated CRC [45]. Adagrasib monotherapy showed an objective RR of 22% and a disease control rate (DCR) of 87%, while the combination of adagrasib and cetuximab yielded even more encouraging results (RR 43% and DCR 100%). This outcome is comparable to that reported in the phase 1b CodeBreaK101 trial, where sotorasib combined with panitumumab in a population of chemorefractory patients showed a DCR greater than 80% and a response rate of 15% [46]. Although KRASG12C mutations identify only a minor percentage (approximately 3–4%) of metastatic CRCs, these patients have a more aggressive disease, poorer survival and are treated with no specific therapy. These results also evidence what was already shown in a preclinical study on the upstream EGFR pathway dependence when the MAPK pathway is inhibited downstream in KRAS-mutant CRC. Studies with adagrasib and sotorasib as second-line treatment are currently ongoing, and will help to better understand the therapeutic sequence of treatment lines among patients with KRASG12C mutant CRC. Sotorasib in combination with an inhibitor of MAPK signaling is being investigated [47].

KRASG12C inhibitors are also being evaluated in combination with immunotherapy, i.e., anti-programmed cell death protein 1 (PD-L1) (Table 1). Preclinical data on sotorasib were recently presented, with the impact of KRASG12C inhibition on immune surveillance in vivo being reported [43]. A syngeneic tumor cell line apposite for testing sotorasib in combination with checkpoint inhibitors was used and characterized in vitro and in vivo; sotorasib caused colon cancer regression in mice xenografts when given in combination with checkpoint inhibitors [48]. Preclinical data showed an increased number of CD3+ T cells and CD8+ T cells after sotorasib exposure, which were further augmented when sotorasib was combined with a PD-L1 immune checkpoint inhibitor. In a preclinical study, sotorasib also prompted a pro-inflammatory microenvironment characterized by enhanced interferon signaling, increased chemokine concentration, antigen processing, natural killer and cytotoxic T cells activity, and markers of innate immune reaction, that were significantly higher compared to MEK inhibition [43]. The ongoing phase 1/2 study of sotorasib is evaluating the combination of sotorasib with PD1/programmed death-ligand 1 (PD-L1) inhibitors (NCT03600883).

Other KRASG12C covalent inhibitors are in phase 1/2 clinical trials (Table 1). These molecules require KRASG12C in the GDP-bound state, prevent SOS-catalyzed nucleotide exchange and impede KRASG12C connection with RAF. So far, different molecules have been detected that bind both to the GDP- and GTP-bound state of KRAS [49,50].

As depicted in Table 1, distinct strategies to target KRAS are ongoing, including anti-KRAS engineered T-cell receptor drugs (i.e., NCT 03745326) and combination therapies with the upstream pathway of SHP2 inhibitors (i.e., NCT03989115 and NCT03114319). RM-007 and RM-008 are compounds that covalently bind KRASG12C and KRASG13C in the GTP-bound state and prevent mutant KRAS from binding SOS and RAS-binding-domain (RBD)-containing effector proteins [11]. These molecules could potentially prevent the occurrence of mechanisms of resistance to GDP-bound KRASG12C covalent inhibitors by targeting the active GTP-bound state.

### 4.2. Strategies to Target KRAS Indirectly or Related Pathways

Blocking one of the crucial stages of RAS activation can indirectly inhibit the RAS pathway.

#### 4.2.1. Inhibitors of the Nucleotide Exchange Cycle

BAY-293, a small-molecule inhibitor that hampers the SOS1–KRAS interaction, was recently identified [51]. BAY-293 shows synergistic growth-inhibitory activity when combined with the KRASG12C covalent inhibitor ARS-853 in KRASG12C cell models. This observation indicates that SOS1 inhibitors might be used in combination with KRASG12C inhibitors that bind to the GDP state, since SOS1 inhibition increases KRASG12C in GDP-bound states. A SOS1 inhibitor, BI-1701963, is currently in a phase 1 clinical trial as a single agent and in combination with the MEK inhibitor trametinib (NCT04111458).

SHP2 is a non-receptor protein tyrosine phosphatase that is essential for the activation of the MAPK pathway [52]. Similarly to a SOS1 inhibitor, blocking of SHP2 prevents the loading of GTP on RAS. Therapy with RMC-4550, a potent and selective SHP2 allosteric inhibitor, decreased cell proliferation in preclinical models and this influence was evident in cells mutant at codon 12, but not at codon 13 or 61. Furthermore, the greatest sensitivity was detected in KRASG12C-mutant cells compared with KRASG12D- or KRASG12V-mutant cells. These results underline how the biological properties of each mutation influence RAS activity’s dependency on guanine exchange, and how mutations with high intrinsic or GAP-mediated hydrolysis are mainly sensitive to SHP2 inhibition. Phase 1/2 trials of SHP2 inhibitors are currently ongoing but no results have been presented (Table 1).

#### 4.2.2. Inhibitors of KRAS Processing and Dimerization

RAS location to the cell membrane is the initial step of RAS activation and requires multiple enzymatic post-translational processing steps; particularly, protein farnesylation by farnesyltransferase (FTase) or geranylgeranylation by geranylgeranyltransferase (GGTase) are enzymatic post-translational modifications that are necessary for RAS activation [53]. Deltarasin and NHTD, small molecules that bind to the farnesyl-binding pocket, prevent KRAS binding and hinder mutant KRAS cells’ growth in vitro and in vivo [54,55]. Unfortunately, FTase inhibitors (FTIs) have shown clinically disappointing results in KRAS-mutant cancers [56,57,58,59], possibly related to the functional redundancy between FTase and GGTase [60]. To elude FTIs’ compensation by GGTase, it is key to target RAS downstream processing enzymes, i.e., isoprenylcysteine carboxyl methyltransferase (ICMT), which are required in KRAS-mutant cells [61], thus providing mutant selectivity and decreasing FTI toxicity. Two small-molecule inhibitors of ICMT (cysmethynil and UCM-1336) impaired RAS membrane localization and reduced cellular growth of RAS-mutant cell lines [62,63,64].

RAS can form dimers on membrane surfaces, a process known as RAS dimerization, that may enhance proteins’ scaffolding and signaling activation [65,66]. The α4–α5 interface is important for RAS dimerization and signaling and can be inhibited by a synthetic binding protein, NS1 [67]. NS1 inhibits HRAS and KRAS dimerization by binding the α4–α5 interface, thus hampering activation of downstream signals and cell growth, without altering RAS localization or GTPase activity [67,68].

A distinct mechanism of RAS inhibition, membrane occlusion, represents a different approach to affect RAS protein–protein interactions and prevent all downstream RAS-driven signaling pathways efficiently. In membrane occlusion, direct interactions between RAS and the lipid membrane closet the RAS-effector-binding interface [69]. The small molecule Cmpd2 prevents KRAS signaling by concurrently binding to a shallow pocket on KRAS and the lipid membrane. Thus, Cmpd2 stabilizes KRAS in an orientation in which its effector-binding site is occluded, which impedes the interaction and the triggering of RAF kinase [69].

#### 4.2.3. Targeting the RAS Pathway

Two different approaches may be useful to target the RAS pathway: detecting genes that are synthetically lethal in RAS-mutated cancer cells, or targeting the tyrosine kinase receptors (EGFR family) and RAS effector pathways, i.e., MAPK and PI3K.

The EGFR-directed antibodies cetuximab and panitumumab in combination with standard chemotherapy are part of the established first or further lines therapies for advanced CRC. Nevertheless, EGFR membrane expression assessed by immunohistochemistry has never been correlated with treatment outcome [70]. Instead, alterations in the EGFR downstream signaling pathway may affect the efficacy of EGFR inhibition as in case of *KRAS* driver mutations [71]. Thus, validation of *RAS* wild-type status is mandatory to establish the treatment approach in advanced CRC [31]. Current guidelines recommend extended *RAS* testing investigating exons 2, 3 and 4 (codons 12, 13, 59, 61, 117 and 146) and only patients showing pan-*RAS* wild-type status should be treated with EGFR inhibitors [5].

The efficacy of the EGFR inhibitors cetuximab and panitumumab in metastatic CRC has been related to specific codon mutations. For example, patients with KRASG13D-mutated CRC treated with cetuximab showed improved progression-free and overall survival compared with other *KRAS* mutations [72]. Conversely, KRASG13D-mutant CRC treated with panitumumab did not show any significant benefit from treatment [73]. Results from the phase 2 clinical trial ICECREAM (ACTRN12612000901808) will clarify the usefulness of cetuximab for metastatic CRC patients with KRASG13D mutations [74].

*RAS* mutation is an early event in carcinogenesis; accordingly, the mutational status is mostly concordant between the primary lesion and metastatic sites [75]. Based on the latest data, this concept was argued against in favor of a fluctuation model, underscoring the role of clonal evolution in influencing tumor responsiveness to target therapy. Pan-*RAS* wild-type tumors may acquire *RAS* mutations during lines of treatment; a hypothesis is that the selective pressure under EGFR inhibitors may promote pre-existing or newly emerging *RAS* mutant clones that are insensitive to therapy [76]. *RAS* mutant clones can arise in formerly *RAS* wild-type colon cancers during the sequence of anti-EGFR treatment, preceding acquired resistance [77,78]. Recent data also indicate that *RAS* analysis among treatment lines could support treatment choices and prove anti-EGFR rechallenges [76]. However, standard recommendations or regulatory agencies do not contemplate the possibility of a rechallenge with EGFR inhibitors [31].

*RAS* mutational status changes subsequent to applied treatment are possibly among the clearest paradigm of genomic, environmental and immune relationships that influence disease progression and growth [79,80]. Gazzaniga et al. [81] showed that 5 out of 11 patients with *RAS*-mutated metastatic CRC previously treated with bevacizumab-containing regimens and analyzed for *RAS* status upon progression by liquid biopsy showed negative tests. The treatment with EGFR inhibitors showed clinical benefit in this group, indicating that formerly *RAS*-mutated tumors reverted to wild-type status upon treatment with VEGF. Probably, *RAS* mutant cells are more susceptible to changes in the vascular supply derived from antiangiogenetic targeted-therapy.

The complex genetic interactions of oncogenic *KRAS* mutations are allele- and tissue-specific and may influence individual responses to targeted drugs [82]. Patients with KRASG12D solid tumors may benefit from treatment with the FDA-approved pan-ERBB inhibitors, afatinib or neratinib, in rationally planned sequential strategies [83,84,85]. A phase 1/2 clinical trial is assessing the effectiveness of neratinib in *RAS*-mutant solid tumors in combination with the histone deacetylase inhibitor divalproex sodium (NCT03919292; Table 1). Pan-ERBB and EGFR inhibitors have a potential role in combination with KRASG12C covalent inhibitors [10]. The inhibition of the EGFR pathway with erlotinib or gefitinib decreases KRASG12C-GTP levels and, therefore, enhances the activity of the KRASG12C covalent inhibitor ARS-853 that interacts with GDP-bound mutant KRAS [86,87].

Active GTP-bound RAS stimulates RAF dimerization and phosphorylation, which prompts RAF kinase activity and phosphorylates RAF substrates MEK1 and MEK2. The signaling cascade continues with MEK phosphorylation of ERK1 and ERK2, which activate several transcription factors, including members of the ETS family, and regulate negative feedback loops [88]. To treat *RAS*-mutant tumors successfully, the MAPK pathway must be nearly completely inhibited. Nowadays, approved BRAF-V600 inhibitors, i.e., dabrafenib and vemurafenib, cannot be employed for RAS-mutant tumors. Moreover, these inhibitors paradoxically activate the MAPK pathway in RAS-mutant cancers by binding to wild-type RAF, prompting RAF dimerization and the downstream phosphorylation of MEK and ERK [89,90]. Pan-RAF inhibitors, i.e., belvarafenib (NCT02405065, NCT03118817; Table 1), are currently under evaluation in phase 1 clinical trials for the treatment of *RAS*-mutant tumors. Belvarafenib monotherapy showed anti-tumor activity in patients with advanced solid tumors harboring *BRAF* and *NRAS* mutations [91].

Overlapping feedback mechanisms sustain the crosstalk between PI3K and MAPK signaling. The inhibition of one pathway can direct the compensatory stimulation of the other; hence, the inhibition of MAPK and PI3K is a promising strategy [92,93]. In vivo studies suggested that inhibitors of the PI3K pathway may be efficacious in *KRAS*-mutant tumors when combined with MEK inhibitors [94,95]. Still, in clinical trials, the combination of these inhibitors had limited tolerability and activity [96,97,98]. To overcome this issue, additional studies tried to select different tyrosine kinases that could inhibit PI3K, MAPK or both signaling pathways. For example, the insulin-like growth factor 1 receptor (IGF1R) triggers downstream signaling pathways in *KRAS*-mutant models, including PI3K-AKT-mTOR and RAS-MEK-ERK [99,100]. The combined inhibition of IGF1R and MEK showed synergistic activity in KRAS-mutant CRC and NSCLC cell lines [99,100]. Additionally, the combination of the KRASG12C covalent inhibitor with an IGF1R inhibitor, linsitinib, showed an enhanced efficacy and tolerability in preclinical models [101]. The design of novel combination treatments to enhance the effect of the KRASG12C inhibitors will require clinical trials.

### 4.3. Evolving Strategies

#### 4.3.1. RNA-Based Approaches to Target KRAS

The efficacy of nanoparticles containing KRAS-targeting small interfering RNA (siRNA) has been tested in mouse models and the results suggest that targeting *KRAS*-mutant cells through specific siRNA might be effective for oncogene silencing and inhibition of tumor growth. Unfortunately, AZD4785, a modified antisense oligonucleotide, which drastically reduced KRAS levels in preclinical models, failed to reduce KRAS levels in a clinical trial (NCT03101839; Table 1). siG12D-LODER, a siRNA against KRASG12D, has shown promising effects in a phase 1 trial in patients with locally advanced pancreatic cancer [102].

#### 4.3.2. Autophagy

KRAS suppression and ERK inhibition have been shown to increase autophagy in mouse models [103,104]. Hydroxychloroquine is an inhibitor of autophagy that is approved by the FDA for the treatment of malaria and is currently being investigated in a phase 1 clinical trial in combination with trametinib for advanced pancreatic cancer patients.

#### 4.3.3. Immunotherapy

Cancer cells evade immune system detection through negative regulatory antigens (checkpoints). They comprise cytotoxic T lymphocyte protein 4 (CTLA4), PD-1 and its ligand PD-L1. CTLA4 is a co-receptor on T cells that negatively regulates T cell activation. PD-L1 expressed on tumor stroma interacts with PD-1 of T cells and generates an intracellular inhibitory signal [105].

CRC generally shows low immunogenicity due to low mutational burdens and tumor-immunosuppressive microenvironments [105,106,107,108]. Expectedly, current immunotherapeutic approaches showed no responses in these tumors [109]. Conversely, durable and deep tumor responses were observed in CRC patients with microsatellite instability-high/mismatch repair-deficient (MSI-H/dMMR) tumors [110,111,112] and pembrolizumab has been approved for the treatment of MSI-H solid malignancies while nivolumab has been approved for MSI-H or dMMR CRC.

Preclinical and clinical data on the targeting of this pathway in *KRAS* mutant tumors indicate that combination treatment of immunotherapies with allele-specific RAS inhibitors might enhance the anti-tumor efficacy of immunotherapy in *RAS*-mutant tumors. Interestingly, *KRAS*-mutated cancer cells can upregulate PDL1 expression levels, which can be reversed with a selective KRASG12C inhibitor (ARS-853) [113]. In addition, in immune-competent mice, treatment with sotorasib resulted in a pro-inflammatory microenvironment, and synergistic activity with immune checkpoint inhibitors [43]. The combination of KRAS inhibitors and anti-PD1 or anti-PDL1 for the treatment of solid tumors is being investigated (NCT03600883; NCT04000529; Table 1).

A second immunotherapeutic approach to treat KRAS-driven tumors encompasses the employment of the adoptive transfer of T lymphocytes expanded ex vivo [114]. For example, the infusion of CD8+ cells targeting mutant *KRAS* mediated the effective regression of metastatic colon cancer that expressed mutant KRASG12D [115]. T-cell receptors (TCRs) characterize a direct method of producing unlimited T cells against a key driver mutation [116]. Currently, different trials are employing this technology to transduce human peripheral blood lymphocytes with the murine TCRs against RASG12D or RASG12V (NCT03745326, NCT03190941; Table 1).

A third immunotherapeutic strategy to treat *RAS*-mutant malignancies uses recognized *RAS*-mutant tumor antigens to stimulate T cell response by vaccination. One of these approaches employs a messenger RNA (mRNA) that encodes neo-epitopes for selected *KRAS* mutations [117]. A phase 1 clinical trial is ongoing to assess the safety and tolerability of mRNA-5671/V941 vaccination in patients with *KRAS* mutations, as a monotherapy or in combination with pembrolizumab (NCT03948763; Table 1).

## 5. Correlation between Genetic Profile and Tumor Staging

Assuming the high prevalence, in stage II and III, of microsatellite instability (MSI) in KRAS-mutated colon cancer, the evaluation of both *BRAF* and *KRAS* mutations in MSI colon cancers is useful for prognosis. *KRAS* mutation is associated with worse prognosis in stage III or high-risk stage II colon cancer patients treated with adjuvant FOLFOX [118]. MSI was found to be associated with favorable survival in early stage CRC compared to MSS CRC. Data highlight that BRAF V600E mutation is associated with worse survival in MSS CRC while the prognostic value of KRAS mutations in both MSS and MSI CRC remains unclear [119].

TNM staging alone does not predict outcomes in CRC patients who might be eligible for adjuvant chemotherapy. The prognostic role of BRAF and KRAS mutations of non-metastatic CRC patients (stage II and III) is still controversial, especially when comparing MSI and MSS tumors. The molecular profile and MSI could be useful as a predictive factor in stage I–III CRC. MMR assays could identify patients with a low risk of recurrence. *KRAS* mutation could guide the use of chemotherapy [120,121,122]. In patients with stage I–III colon cancer, laterality, *KRAS* mutation and microsatellite instability status were not independently prognostic after curative resection [123]. Next-generation sequencing screens multiple mutations in multiple genes simultaneously without the need to perform several sequential tests. This highlights its superiority in terms of sensitivity and specificity over other techniques. The detection of KRASG12C has opened up new scenarios.

Although KRASG12C was identified as a potential drug target and predictor of responses to the novel sotorasib target treatment, clinical mechanisms of resistance remain unknown [124].

Several mechanisms have been proposed to explain the acquired resistance to KRASG12C inhibitors. Fedele et al. [125] found that G12C evokes adaptive resistance in vitro and in vivo by inducing KRASG12C re-activation. Ryan et. al. [126] argued that the adaptive resistance to G12C involves the upregulation of RTK signaling and the activation of WT RAS. Xue et al. [127] reported that resistance arises from pre-existing heterogeneity that enables some tumor cells to survive by inducing mutant KRAS to levels that exceed the inhibitor-targeting capacity. It was also shown that the tumor microenvironment (TME) regulates cancer resistance to therapies. The adaptive resistance to cancer treatment driven by the TME might play a crucial role in tumor recurrence and metastasis [128]. Moreover, epigenetic mechanisms, such as methylation, regulate important signaling pathways involved in therapy resistance in colon cancer as well [129].

The factors that can affect treatment response are many and their interaction can induce an adaptative resistance. Potential mechanisms of resistance are complex and include genomic mechanisms such as secondary *KRAS* mutations (i.e., G12D, Y96C and H95R), amplifications of KRASG12C and MET, as well as histologic mechanisms such as transformation to other histotypes. Awad recently claimed that “novel combinatorial strategies will be necessary to delay or overcome resistance in KRASG12C-mutant cancers” in the New England Journal of Medicine [130]. This new scenario opens the discussion on the need to study the molecular structure of CRC in the various steps, at diagnosis and during follow-up and progression.

Data in the literature suggest that the tumor, especially when treated with chemo- and possibly radiotherapy, can acquire new mutations (tumor mutational burden—TMB) and be responsive to immunotherapy [131]. The study of the tumor to investigate its dynamism would require a re-biopsy; however, a patient’s performance status often does not allow it or it is not technically feasible. Liquid biopsy might help us. The non-invasive study of mutational heterogeneity can be carried out as an alternative when the sample is not sufficient or as an addition to known information.

Tumor tissue is characterized by areas with different genetic profiles [132]. Tissue genotyping on samples obtained by tissue biopsy might not detect all actionable mutations present in the tumor due to the limits of sampling. The tissue expresses information restricted to the area of the tumor in which the biopsy is performed. Inability to perform the sampling from all possible metastases is another issue to take into account. Recently, a blood-based liquid biopsy approach for monitoring CRC has emerged and is about to enter clinical practice [133]. Using a liquid biopsy-based approach, the pathology laboratory can extract tumor DNA from a blood sample, which can be used for detecting cancer at an early stage and for follow-up of disease progression. Tumor biopsy represents the photograph of the tumor at a precise moment. The purpose of liquid biopsy is to monitor the tumor, which might make it possible to understand its evolution, to characterize the molecular profile of CRC, and to identify therapeutic targets and mechanisms of drug resistance with a non-invasive and more sustainable methods, in economic terms, at any time. Moreover, during follow up, it could detect a change in tumor histology under selective pharmacological pressure. More studies on colon cancer are needed in order to solve related problems, such as the low amounts of CTCs and ctDNA in samples [134,135,136]. For example, in lung cancer, the possibility of acquiring a post-therapy neuroendocrine phenotype is widely documented [137,138,139].

The personalization of treatment for each patient with CRC with metastases aims to obtain the maximum advantage, in terms of effectiveness, from the administration of the target drugs. The determination of KRAS is essential because the state of the KRAS protein can influence the prognosis of the disease and the response. The response to targeted therapy aims at the inactivation of EGFR. In addition, KRAS mutation status may predict time to progression.

## 6. Future Viewpoints

Early data on KRAS inhibitors seem encouraging. KRAS, specifically G12C, is no longer an “undruggable target”, but rather, it is a valuable bullet among targetable molecular alterations. Not only should forthcoming studies evaluate the effectiveness of covalent KRASG12C inhibitors in the treatment of KRASG12C-mutated cancers, but they should also aim to recognize tumors that are likely to gain benefit from monotherapy as well as tumors that will likely benefit from rational combination approaches.

The question of whether the more limited activity of KRASG12C inhibitors in CRC compared to NSCLC is related to the earlier emergence of multiple acquired resistance mechanisms or to the existence of adaptive signaling pathways will need intensified research and supports the basis for ongoing clinical trials of adagrasib (NCT04330664) or sotorasib (NCT04185883) in combination with inhibitors of RTKs or SHP2.

Finally, the variety of on-target and off-target mechanisms that can confer resistance to KRASG12C inhibitors supports the development of other KRAS inhibitors with alternative types of binding and distinct allele specificity. The improvement of effective combination regimens will be the prerequisite against resistance mechanisms that may develop during treatment.

## Figures and Tables

**Table 1:** A selection of clinical trials with KRAS, SHP2 and SOS1 inhibitors registered on ClinicalTrials.gov, accessed on 6 March 2022.

ClinicalTrials.gov Identifier	Agent(s)	Phase Study	Setting
NCT03600883	Sotorasib (KRASG12 inhibitor)	½	Advanced solid tumors with KRASG12C mutation
NCT03785249	Adagrasib (KRASG12 inhibitor)	½	Advanced solid tumors with KRASG12C mutation
NCT05162443 *	Adagrasib (KRASG12 inhibitor)	-	Advanced solid tumors with a KRAS G12C mutation
NCT05263986	Adagrasib (KRASG12 inhibitor)	1	Chinese patients with advanced solid tumor with KRAS G12C mutation
NCT04975256	Adagrasib (KRASG12 inhibitor) + BI 1701963 (pan-KRAS SOS1 inhibitor)	1	Advanced solid tumors with KRASG12C mutation
NCT04006301	JNJ-74699157 (KRASG12 inhibitor)	1	Advanced solid tumors with KRASG12C mutation
NCT04165031	LY3499446 (KRASG12 inhibitor)	1/2	Advanced solid tumors with KRASG12C mutation
NCT04585035	D-1553 (KRASG12 inhibitor)	1/2	Advanced solid tumors with KRASG12C mutation
NCT04956640	LY3537982 (KRASG12 inhibitor) alone or in combination	1/2	Advanced solid tumors with KRASG12C mutation
NCT05194995	JAB-21822 (KRASG12 inhibitor) + cetuximab (EGFR inhibitor)	1/2	Advanced CRC and other solid tumors with KRASG12C mutation
NCT05002270	JAB-21822 (KRASG12 inhibitor) alone and combination with cetuximab (EGFR inhibitor)	1/2	Advanced solid tumors with KRASG12C mutation
NCT04973163	BI 1823911 (KRASG12 inhibitor) alone and combined with other anti-cancer therapies	1	Advanced solid tumors with KRASG12C mutation
NCT05005234	GFH925 (KRASG12 inhibitor)	1/2	Advanced solid tumors with KRASG12C mutation
NCT04793958	Adagrasib (KRASG12 inhibitor) + cetuximab (EGFR inhibitor) versus chemotherapy	3	Second-line treatment setting in patients with CRC with KRASG12C mutation
NCT05178888	Adagrasib (KRASG12 inhibitor) + palbociclib (CDK4/6 inhibitor)	1	Advanced solid tumors with KRASG12C mutation
NCT05010694	GH35 (KRASG12 inhibitor)	1	Advanced solid tumors with KRASG12C mutation
NCT04185883	Sotorasib (KRASG12 inhibitor) +/− Anti-cancer therapies	1/2	Advanced solid tumors with KRASG12C mutation
NCT05198934	Sotorasib (KRASG12 inhibitor) + panitumumab (EGFR inhibitor) vs. investigator’s choice (trifluridine and tipiracil, or regorafenib)	3	Previously treated metastatic KRASG12C-mutated CRC
NCT04449874	GDC-6036 (KRASG12 inhibitor) alone or in combination	1	Advanced solid tumors with KRASG12C mutation
NCT04699188	JDQ443 (KRASG12 inhibitor) alone or in combination	1/2	Advanced solid tumors with KRASG12C mutation
NCT05009329	JAB-21822 (KRASG12 inhibitor)	1/2	Chinese patients with advanced solid tumor with KRAS G12C mutation
NCT04678648	RSC-1255 (pan-mutant and wild-type RAS inhibitor)	1	Advanced solid tumors
NCT03745326	Anti-KRASG12D murine T-cell receptor(mTCR) peripheral blood lymphocytes (PBL)	1/2	HLA-A*11:01 positive patients with advanced solid tumors expressing G12D-mutated RAS
NCT03190941	Anti-KRASG12 V mTCR PBL	1/2	HLA-A*11:01 positive patients with advanced solid tumors expressing G12V-mutated RAS
NCT 03114319	TNO155 (SHP2 inhibitor)	1	Advanced EGFR mutant NSCLC, KRASG12 mutant NSCLC, Esophageal Squamous Cell Cancer (SCC), Head/Neck SCC, Melanoma
NCT03634982	RMC-4630 (SHP2 inhibitor)	1	Advanced relapsed/refractory solid tumors
NCT03518554	JAB-3068 (SHP2 inhibitor)	1	Advanced solid tumors
NCT04721223	JAB-3068 (SHP2 inhibitor) in combination with PD1 inhibitor	1/2	Advanced solid tumors
NCT03565003	JAB-3068 (SHP2 inhibitor)	1/2	Advanced solid tumors in China
NCT04121286	JAB-3312 (SHP2 inhibitor)	1	Advanced solid tumors in China
NCT04045496	JAB-3312 (SHP2 inhibitor)	1	Advanced solid tumors
NCT04720976	JAB-3312 (SHP2 inhibitor) in combination with other agents	1/2	Adult patients with advanced solid tumors
NCT04111458	BI 1701963 (pan KRAS/SOS1 inhibitor) +/− Trametinib (MEK inhibitor)	1	Advanced solid tumors with KRAS mutation
NCT04627142	BI 1701963 (pan KRAS/SOS1 inhibitor) + Irinotecan	1	Unresectable locally advanced or metastatic KRAS mutant CRC
NCT04835714	BI 1701963 (pan KRAS/SOS1 inhibitor) alone and in combination with BI 3011441 (MEK inhibitor)	1	Advanced solid tumors with KRAS mutation
NCT04330664	Adagrasib (KRASG12 inhibitor) + TNO155 (SHP2 inhibitor)	1/2	Advanced solid tumors with KRASG12C mutation
NCT03989115	RMC-4630 (SHP2 inhibitor) + Cobimetinib (MEK inhibitor)	1/2	Relapsed/refractory solid tumors with specific genomic aberrations (KRAS mutations and amplifications, BRAF class 3 mutations, or NF1 LOF mutations)
NCT04916236	RMC-4630 (SHP2 inhibitor) and LY3214996 (ERK1/2 inhibitor)	1	Advanced or metastatic *KRAS* mutant NSCLC, CRC or pancreatic adenocarcinoma
NCT04418661	SAR442720 (SHP2 inhibitor) in combination with other agents	1/2	Advanced solid tumors
NCT04252339	RLY-1971 (SHP2 inhibitor)	1	Advanced solid tumors
NCT04800822	PF-07284892 (SHP2 inhibitor) alone or in combination with other agents	1	Advanced solid tumors
NCT04528836	BBP-398 (SHP2 inhibitor)	1	Advanced solid tumors
NCT04843033	SH3809 (SHP2 inhibitor)	1	Advanced solid tumors in China
NCT04670679	ERAS-601 (SHP2 inhibitor) alone or in combination with cetuximab (EGFR inhibitor)	1	Advanced solid tumors
NCT03919292	Neratinib (pan-ErbB inhibitor) and divalproex sodium (HDAC inhibitor)	1/2	Advanced solid tumors and RAS-mutated cancers
NCT02405065	HM95573 (pan-RAF kinase inhibitor)	1	Solid tumors
NCT03118817	HM95573 (pan-RAF Kinase Inhibitor)	1	BRAF, KRAS or NRAS mutant solid cancers
NCT03101839	AZD4785 (KRAS antisense oligonucleotide)	1	Advanced solid tumors with *KRAS* mutations
NCT04000529	TNO155 (SHP2 inhibitor) + Spartalizumab (anti-PD1 monoclonal antibody) or Ribociclib (CDK 4/6 inhibitor)	1	Advanced solid tumors
NCT03948763	mRNA-5671/V941 +/− Pembrolizumab (anti-PD1 monoclonal antibody)	1	Advanced or metastatic *KRAS* mutant NSCLC, CRC or pancreatic adenocarcinoma

* Expanded access.

## Data Availability

Not applicable.
